# Retrospective Analysis of Ventriculitis in External Ventricular Drains

**DOI:** 10.1155/2018/5179356

**Published:** 2018-09-02

**Authors:** Stephen Albano, Blake Berman, Glenn Fischberg, Javed Siddiqi, Bolin Liu, Yasir Khan, Atif Zafar, Syed A. Quadri, Mudassir Farooqui

**Affiliations:** ^1^Desert Regional Medical Center, Palm Springs, CA, USA; ^2^University of New Mexico, NM, USA

## Abstract

**Background:**

Nosocomial EVD-related ventriculitis is a major complication and a significant cause of morbidity and mortality in critically ill neurological patients. Questions remain about best management of EVDs. The purpose of this study is to compare our incidence of ventriculitis to studies using different catheters and/or antibiotic coverage schemes and determine whether c-EVD with prolonged antibiotics given for the duration of drain placement is inferior to ac-EVD with pp-abx or ac-EVD with prolonged antibiotics for prevention of ventriculitis.

**Methods:**

A retrospective chart review of all patients who had EVDs placed from January 2010 through December 2015 at home institution was performed. Statistical analysis was performed using Fisher's exact test to compare incidence of ventriculitis identified in other studies with that of home institution.

**Results:**

The study included 107 patients, 66 (61.7%) males and 41 (38.3%) females. Average age was 56 years ranging from 18 to 95 years. Average length of drain placement was 7.8 days ranging from 2 to 23 days. Average length of drain placement in infected drains was 13.3 days ranging from 11 to 15 days. There were 3 cases with positive CSF cultures (*Staphylococcus haemolyticus* and* Staphylococcus epidermidis* x 2). There were 2 cases with a CSF having a positive gram stain but failed to yield any bacterial growth on culture and did not meet predefined criteria.

**Conclusions:**

The c-EVD with prolonged antibiotics given for the duration of drain placement is not inferior to ac-EVD with pp-abx or ac-EVD with prolonged antibiotics for prevention of ventriculitis. The c-EVD with prolonged antibiotics is superior to c-EVD with pp-abx and conventional EVD without antibiotics for prevention of ventriculitis. Selection should include considerations for antibiotic stewardship and cost effectiveness. Future studies should also utilize clinical and CSF profile criteria in addition to positive CSF cultures for identifying ventriculitis to prevent line colonization from classification as ventriculitis in analysis.

## 1. Background

External ventricular drains (EVD) are often placed for decompression in cases of trauma, hemorrhage, mass effects, and cerebral edema or to monitor intracranial pressure (ICP). Since first attempted in the 18th century by Claude-Nicholas Le Cat, the insertion of an EVD is perhaps one of the most commonly performed neurosurgical procedures in neurologic intensive care units today [1, 2]. The current research in EVDs has focused on improving the overall safety of the procedure, which includes development of guidance-based systems, virtual reality simulators for training, and antibiotic-impregnated catheters.

Like every intervention, EVDs have risks, and one of the more serious risks is a ventricular catheter related infection which can manifest as ventriculitis [2–10]. The current reported rates of ventriculitis range from 0% to 45% [3–9, 11–15]. In an attempt to prevent such complications various strategies have been employed. These strategies can include prophylactic antibiotics for the duration of the drain placement, in which a previous randomized study demonstrated reduction in ventriculitis [16]. Conversely some studies report similar incidences of ventriculitis in patients receiving prophylactic antibiotics for the duration of EVD versus placebo [11]. Studies have also demonstrated that the use of prolonged systemic antibiotic prophylaxis in the setting of EVD placement yielded a higher rate of nosocomial infection [3]. This raises a concern that the use of prophylactic antibiotics to prevent ventriculitis could lead to more resistant nosocomial infections.

In an attempt to find alternatives for prolonged prophylactic antibiotics, silver impregnated and antibiotic coated external ventricular drain (ac-EVD) catheters have been developed. Studies have shown that patients with EVDs using silver impregnated catheters developed less ventriculitis than patients with conventional external ventricular drain (c-EVD) catheters [6]. Similarly ac-EVDs have demonstrated decreased incidences of ventriculitis compared to standard catheters [13]. However the utility of ac-EVD has not been definitively proven as demonstrated by the randomized controlled trial by Pople et al. involving 434 patients which failed to show a reduction in the incidence of ventriculitis with the use of ac-EVD catheters compared to c-EVD [15]. Given the opposing data, questions remain about how best to manage EVDs. Therefore, the purpose of this study was to define the rate of ventriculitis at the home institution under practices used from January 2010–December 2015 and compare incidence to studies using different catheters and/or antibiotic coverage schemes.

## 2. Methods

A retrospective chart review of all patients who had EVDs placed from January 2010 through December 2015 at home institution was performed to assess for ventriculitis. Ventriculitis for this study was defined as bacterial organism identified from cerebrospinal fluid (CSF) on 2 of 2 cultures, or gram stain with temperature >38C, and CSF pleocytosis (>100WBC/uL), elevated protein (>50mg/dL), and decreased CSF glucose (<40% serum glucose).

Inclusion criteria for this study included any patient 18 years of age or older who had an EVD placed at the home institution. The exclusion criteria included any prior violation of cranial bones, concurrent craniotomy or craniectomy, concurrent intracranial device (licox, subdural drain), pregnant, drain in place less than 48 hours, or death within 48 hours of placement.

Data recorded included age of the patient, gender, race, antibiotics used, duration of drain, bacteria cultured, bacterial resistances, indication for EVD, intraventricular hemorrhage (IVH), vasospasm, organism and resistances of nosocomial pneumonia and/or blood stream infection (BSI), clostridium difficile, CSF studies, year placed, administration of intrathecal (IT) tPA, and allergies to antibiotics.

Aseptic technique was used during placement of c-EVDs. Prophylactic antibiotic coverage was left to the discretion of the attending physician; however in general cefazolin 1 gram IV every 8 hours with first administration approximately one hour prior to placement of EVD and continued until EVD was removed. Antibiotic coverage was altered at physician discretion if patient had allergies or developed signs or symptoms concerning for concurrent infection. CSF samples were obtained via ventriculostomy when there was clinical suspicion for ventriculitis.

EVD placement was identified in patient records utilizing ICD code 02.21. The search returned 258 patient records containing this code from January 2010–December 2015. Of these, 3 patients were excluded due to no record of actual insertion of an EVD, 3 were excluded for ageless than 18 years, 69 patients were excluded for prior or concurrent craniotomy, 36 patients were excluded for prior or concurrent craniectomy, 5 patients were excluded for concurrent licox placement, 4 patients were excluded for concurrent burr holes to perform endoscopic third ventriculostomy, 7 patients were excluded for concurrent subdural drain placement, 8 patients were excluded for ventriculoperitoneal (VP) shunt placement, 1 patient was excluded for prior EVD, 1 patient was excluded for cranioplasty, 13 patients were excluded for death within 48 hours of drain placement, and 1 patient was excluded for burr hole aspiration of brain cyst. This left 107 patients to be included for analysis.

## 3. Statistical Analysis

Statistical analysis was performed using Fisher's exact test to compare incidence of ventriculitis in our study versus incidence of ventriculitis in studies using various types of catheters and antibiotic regimens.

## 4. Results

The study included 107 patients, 66 (61.7%) males and 41 (38.3%) females. Average age was 56 years ranging from 18 to 95 years. Race included 52 (48.6%) white, 3 (2.8%) black, and 52 (48.6%) other (predominantly Hispanic). Average length of drain placement was 7.8 days ranging from 2 to 23 days. Average length of drain placement in infected drains was 13.3 days ranging from 11 to 15 days. Indications for EVD (Table 1) included 35 (32.7%) for intracranial hemorrhage (ICH), 28 (26.2%) trauma, 27 (25.2%) aneurysmal rupture, 10 (9.3%) edema, and 7 (6.5%) subarachnoid hemorrhages (SAH).

Fisher's exact test was used to compare retrospective data obtained in current study to that of other studies (Table 2).

The studies shown in Table 2 were also collectively grouped by type of external ventricular catheter and antibiotic regimen and then compared to data collected in current study. This showed a statistically significant difference between c-EVD with prolonged antibiotics and c-EVD without antibiotics and c-EVD with periprocedural antibiotics (pp-abx). There was no statistically significant difference from other studies employing c-EVD with prolonged antibiotics, ac-EVD with pp-abx, or from ac-EVD with prolonged antibiotics.

There were 3 cases with positive CSF cultures (*Staphylococcus haemolyticus* and* Staphylococcus epidermidis* x 2). The antibiotic resistance profiles were different in each case (Table 3). There were 2 cases with a CSF having a positive gram stain but failed to yield any bacterial growth on culture and did not meet predefined criteria (fever >38C, CSF pleocytosis >100WBC/uL, elevated protein >50mg/dL, and decreased CSF glucose <40% serum glucose) for ventriculitis and were therefore not included in infected category to be consistent with other studies for comparison.

Secondary objectives of the study included identifying whether intraventricular hemorrhage (IVH) is correlated with increased incidence of ventriculitis. The results showed IVH in 1 of the 3 (33.3%) ventriculostomies with ventriculitis, and IVH in 64 out of 104 (61.5%) ventriculostomy cases without ventriculitis. The metrics are not sufficient to make any conclusions about correlation between IVH and ventriculitis in patients with ventriculostomies with statistical confidence. Cases with IVH frequently utilized intrathecal (IT) tPA. There were 1 of 3 (33.3%) ventriculostomy cases with ventriculitis that utilized intrathecal (IT) tPA and 26 of 104 (25%) ventriculostomy cases without ventriculitis. The number of cases does not permit statistical analysis that would allow for comparison to determine if there is a correlation between IT tPA and ventriculitis in patients undergoing ventriculostomy.

Of the 107 included patients, 3 patients developed clostridium difficile infections identified by toxin assay, and 37 developed nosocomial pneumonia. Some pneumonia was polymicrobial (that is why the sum of the sputum culture species identified and the 70 negative sputum cultures are greater than 107). Of the nosocomial pneumonia, 6 were resistant to the original antibiotic (possibly representing contamination). The sputum culture profile is shown in Table 4. Of the patient's with ventriculitis, none of the bacteria isolated were found to have a resistance to initial antibiotic given.

## 5. Discussion

Placement of EVD is often necessary in neurological intensive care patients. Since first performed as early as 1744, the history of EVDs has gone through 4 eras of evolution: development of the technique (1850-1908), technological advancements (1927-1950), expansion of indications (1960-1995), and the current era of accuracy, training, and infection control (1995-present) [1]. A major complication of this procedure is development of nosocomial EVD-related ventriculitis, which is a significant cause of morbidity and mortality in critically ill neurological patients [2, 7–9, 19]. The incidence of ventriculitis has been reported in current literature ranging from 0% to 45% [3–9, 11–15, 17].

The current study identified three cases of ventriculitis based on positive CSF culture in 2 of 2 samples to be consistent with other studies. When looking at the current study,* Staphylococcus epidermidis* was identified in 2 of the 3 cases of ventriculitis and* Staphylococcus haemolyticus* in 1 of the 3 cases. The initial antibiotic selected in all the cases included cefazolin, and in all cases the bacterium was not resistant to cefazolin. This becomes suspicious for contamination since antibiotic selected should cover gram positive organisms and the culture did not demonstrate resistance to cephalosporins.

In all the cases of ventriculitis, blood cultures were negative, sputum cultures were negative, and urinalysis (UA) did not support UTI. The CSF profile in terms of WBC, protein, and glucose did not support bacterial meningitis and would therefore be unlikely to have a ventriculitis. However, blood leukocytosis in all cases and fever in 1 of the 3 cases initiated an infectious workup. When blood cultures, sputum cultures, and urinalysis did not show signs of infection, CSF cultures were obtained. Positive CSF cultures with blood leukocytosis without CSF pleocytosis or decrement in CSF glucose could indicate ventricular catheter colonization or possibly early identification of infection which has not yet caused significant shifts in CSF profile.

However, the majority of studies identified ventriculitis by CSF cultures without qualifiers for CSF profile or clinical findings. This shows that studies could have been identifying contaminants or ventricular catheter colonization that were termed ventriculitis based on predefined criteria. This may contribute to the wide range of reported incidences of ventriculitis in literature [3–9, 11–15, 17]. The studies also used various sampling schemes (Table 5) in which manipulation exposes the patient, catheter, and sample to contamination. Given that the definition of ventriculitis affects incidence, future studies should include clinical and/or additional abnormal CSF qualifiers to prevent identifying contaminants or line colonization as ventriculitis.

Our results representing c-EVD with prolonged antibiotics show no statistically significant difference when compared to the 6 of the 8 other studies (corresponding p values in Table 2) that utilized c-EVD catheters with prolonged antibiotics [4–6, 11, 15–18]. This suggests that our incidence of ventriculitis is consistent with other studies. The studies by Wright K et al. and Zambramski JM et al. utilized c-EVD with prolonged antibiotics and reported a statistically significant difference in incidence of ventriculitis [12–14]. This demonstrates that catheter infections are a multifactorial process and extend beyond just catheter type and antibiotic coverage. When our incidence is compared to ac-EVD with pp-abx, there is no statistically significant difference. When comparing our results with ac-EVD in the setting of prolonged antibiotics, there is no statistically significant difference. Our c-EVD with prolonged antibiotics demonstrated a statistically significant difference when compared with c-EVD and pp-abx and in c-EVDs without antibiotics. In an attempt to control for sample sizes, varying local practices and, to arrive at a generalizable conclusion, an aggregate of ventriculitis rates were assembled as shown in Table 2. This shows a statistically significant difference when our c-EVD with prolonged antibiotics is compared to c-EVD without antibiotics and conventional EVD with pp-abx. However, there is no statistically significant difference when compared to ac-EVD with pp-abx or ac-EVD with prolonged antibiotics.

As shown in Table 5, each study had varying CSF sampling patterns. CSF sampling in our study only occurred when there was clinical suspicion of ventriculitis. This could have yielded a smaller incidence of infection in our population when compared to those of other studies which may be why there is no difference when compared to ac-EVD of either antibiotic scheme or mixed differences when compared to conventional EVD's with prolonged antibiotics (same practice in current study).

## 6. Conclusions

The c-EVD with prolonged antibiotics given for the duration of drain placement is not inferior to ac-EVD with pp-abx or ac-EVD with prolonged antibiotics for prevention of ventriculitis. The c-EVD with prolonged antibiotics is superior to c-EVD with pp-abx and conventional EVD without antibiotics for prevention of ventriculitis. Infection control is multifactorial consisting of more than just catheter type and antibiotic coverage. Therefore, in considering which type of EVD to utilize given the similarly performing combinations of catheter type and antibiotic schedules in terms of preventing ventriculitis, selection should include considerations for antibiotic stewardship and cost effectiveness. This would suggest ac-EVD with pp-abx should be used in ventriculostomies to prevent ventriculitis. Future studies should also utilize clinical and CSF profile criteria in addition to positive CSF cultures for identifying ventriculitis to standardize and identify true ventriculitis.

## Figures and Tables

**Table 1:** Demographics and indications for EVD placement n=107 (percentages may not total 100% due to rounding errors).

**AGE**	
Average (years)	56
Range	18-95

**GENDER**	
Male	66 (61.7%)
Female	41 (38.3%)

**RACE**	
White	52 (48.6%)
Black	3 (2.8%)
Other	52 (48.6%)

**INDICATION**	
ICH	35 (32.7%)
Trauma	28 (26.2%)
Aneurysmal rupture	27 (25.2%)
Edema	10 (9.3%)
SAH	7 (6.5%)

**Table 2:** Incidence of ventriculitis in various studies compared incidence of ventriculitis in current study.

Author	c-EVD	p-value (vs Albano SD et al	c-EVD with pp- Abx	p-value (vs Albano SD et al)	c-EVD prolonged ABx	p-value (vs Albano SD et al)	ac-EVD with pp-ABx	p-value (vs Albano SD et al)	ac-EVD prolonged ABx	p-value (vs Albano SD et al)
Albano SD (current study)					3/107 (3%)					

Wong GK [4]					3/94 (3%)	0.31	1/90 (1%)	0.29		

Abla AA [5]							0/64 vs 0/65 (0%)	<0.0001		

Poon WS [5]			12/113 (11%)	0.015	3/115 (3%)	0.32				

Wyler AR [17]	7/26 (27%)	0.004			4/44 (9%)	0.087				

Blomstedt GC [11]	1/27 (4%)	0.42			1/25 (4%)	0.41				

Wright K [12]					12/51 (24%)	<0.0001			2/47 (4%)	0.32

Zabramski JM [14]					13/139 (9%)	0.025			2/149 (1%)	0.25

Pople I [15]					5/181 (3%)	0.29			4/176 (2%)	0.29

Alleyne Jr CH [18]			4/99 (4%)	0.27	8/209 (4%)	0.24				

Aggregate	8/56 (14%)	0.007	16/212 (8%)	0.050	49/858 (6%) (not including data from current study)	0.092	1/219 (<1%)	0.094	8/372 (2%)	0.25

**Table 3:** Profile of ventriculitis cases identified.

	CSF culture	Resistance Profile	Initial Antibiotic	Antibiotic change to	Duration of drain	Indication	IVH	IT tPA
Case 1	Staphylococcus epidermidis	Oxacillin, Penicillin G	Cefazolin	Ceftazidime, Vancomycin, Metronidazole	14 days	ICH	Yes	Yes

Case 2	Staphylococcus epidermidis	Erythromycin, Oxacillin, Penicillin G, Clindamycin	Cefazolin, Amoxicillin/ clavulanate	Ceftazidime, Vancomycin, Levaquin (pneumonia)	15 days	Cerebral edema	No	No

Case 3	Staphylococcus haemolyticus	Erythromycin, Oxacillin, Penicillin G, TMP-SMZ	Cefazolin	Vancomycin, Meropenem, Ceftriaxone (concurrent UTI)	11 days	Aneurysm rupture	No	No

**Table 4:** Sputum cultures for nosocomial pneumonia.

Negative Sputum Culture	70 (65.4%)
Klebsiella pneumonia	6 (5.6%)
Klebsiella oxytoca	1 (0.9%)
Klebsiella planticola	1 (0.9%)
Escherichia coli	1 (0.9%)
Staphylococcus aureus	10 (9.3%)
Pseudomonas aeuroginosa	2 (1.9%)
Pseudomonas putida	2 (1.9%)
Streptococcus pneumonia	3 (2.8%)
Streptococcus mitis	1 (0.9%)
Streptococcus oralis	1 (0.9%)
Enterobacter aerogenes	2 (1.9%)
Enterobacter cloacae	3 (2.8%)
Enterobacter faecalis	2 (1.9%)
Serratia marcesens	3 (2.8%)
Citrobacter koseri	2 (1.9%)

**Table 5:** Outline of trial data, definition for infection, and sampling schedule.

Author	c-EVD	c-EVD with pp- Abx	c-EVD prolonged ABx	ac-EVD with pp-ABx	ac-EVD prolonged ABx	p-value between groups in each respective study	Conclusion	Definition of infection	Sampling rate	Type of study	Time Frame
Albano SD et al			3/107 (3%)					Infection – positive CSF culture with sensitivity	Clinical suspicion	Retrospective review	Jan 2010 - Dec 2015

Wong GK et al [4]			3/94 (3%)	1/90 (1%)		0.282	“Antibiotic impregnated catheters are as effective as systemic antibiotics in the prevention of CSF infection and their corresponding nosocomial infection rates are not statistically different”	Infection – positive CSF bacterial culture with CSF white cell count > 10/mm^3^, CSF protein level > 0.8g/L and CSF to serum glucose ratio <0.4	CSF collected every 5 days on evidence of clinical sepsis	Randomized trial	Apr 2004 - Dec 2008

Abla AA et al [5]				0/64 vs 0/65 (0%)			“These results support the use of antibiotic impregnated EVD catheters in routine clinical practice” due to “comparison with reported mean of nearly 9% for standard EVD catheters”	Infection – positive CSF cultures (same organism grown on two different media or on same medium twice)	CSF collected twice weekly Mondays and Thursdays	Prospective sequential series trial	Jan 2007 – June 2008

Poon WS et al [16]		12/113 (11%)	3/115 (3%)			0.01	“antibiotic regimen against both Gram positive and Gram-negative bacteria was effective in preventing ventriculostomy related sepsis caused by common pathogens”	Infection – CSF culture and/or CSF white cell count >50/mm^3^	Unknown sampling frequency. Catheter changed every 5 days	Randomized trial	Oct 1993 – Sep 195

Wyler AR et al [17]	7/26 (27%)		4/44 (9%)			“chi square and t-tests of significance (with alpha level at 0.05) show statistically different infection rates between the two groups”	“If on the other hand, the anticipated duration of ventriculostomy is more than 3 days or CSF viscosity is usually high, prophylactic antibiotics should be used”	Infection – not defined however organism identified by culture	CSF collected upon insertion and removal	Retrospective review	Jan-1963 – Jan 1969

Blomstedt GC et al [11]	1/27 (4%)		1/25 (4%)			0.51 (p calculated from data reported)	No conclusion specific to antibiotic with EVD as data to the left was part of a secondary outcome measure	Infection – not defined however organism identified by culture	Timing and frequency of CSF sample not described	Double blind randomized trial	Apr 1980 – Jun 1983

Wright K et al [12]			12/51 (24%)		2/47 (4%)	0.0265	“Rates of VRIs (ventriculostomy related infections) have decreased with the addition of ac-EVDs to the routine use of prolonged systemic antibiotics at the author's institution	Infection – clinical signs and symptoms of infection, abnormal CSF parameters and a positive CSF culture	CSF sample when clinically indicated	Retrospective review	Feb 2007 – Nov 2009

Zabramski JM et al [14]			13/139 (9%)		2/149 (1%)	0.002 (chi-square test)	“The use of EVD catheters impregnated with minocycline and rifampin can significantly reduce the risk of catheter related infections”	Infection – positive CSF cultures (same organism on two different media or same medium twice)	CSF collected at time of insertion, every 72 hours and on removal	Multicenter prospective, randomized controlled trial	Dec 1998 – Mar 2001

Pople I et al [15]			5/181 (3%)		4/176 (2%)	1.0	“AI-EVD (antibiotic impregnated) catheters were not associated with risk reduction in EVD infection compared to standard catheters”	Infection – CSF sample demonstrate positive gram stain that was culture positive in agar growth	CSF sample at time of insertion, day 3 post implant, catheter removal, time of suspected infection	Multicenter, international, prospective, randomized open label trial	Nov 2004 – Sep 2010

Alleyne Jr CH et al [18]		4/99 (4%)	8/209 (4%)			0.242 (Fisher exact test calculated using data reported)	“use of continuous prophylactic antibiotics offers no benefit over periprocedural dosing alone”	Infection – positive CSF cultures	CSF sampled at time of insertion and twice weekly	Retrospective review	Jan 1996 – Jun 1997

## Data Availability

The data used to support the findings of this study are available from the corresponding author upon request.
